# Zinc enhances hippocampal long-term potentiation at CA1 synapses through NR2B containing NMDA receptors

**DOI:** 10.1371/journal.pone.0205907

**Published:** 2018-11-28

**Authors:** John A. Sullivan, Xiao-lei Zhang, Arthur P. Sullivan, Linnea R. Vose, Alexander A. Moghadam, Victor A. Fried, Patric K. Stanton

**Affiliations:** 1 Cell Biology & Anatomy, New York Medical College, Valhalla, New York, United States of America; 2 Psychology & Education, Touro School of Health Sciences, New York, New York, United States of America; Bilkent University, TURKEY

## Abstract

The role of zinc (Zn^2+^), a modulator of N-methyl-D-aspartate (NMDA) receptors, in regulating long-term synaptic plasticity at hippocampal CA1 synapses is poorly understood. The effects of exogenous application of Zn^2+^ and of chelation of endogenous Zn^2+^ were examined on long-term potentiation (LTP) of stimulus-evoked synaptic transmission at Schaffer collateral (SCH) synapses in field CA1 of mouse hippocampal slices using whole-cell patch clamp and field recordings. Low micromolar concentrations of exogenous Zn^2+^ enhanced the induction of LTP, and this effect required activation of NMDA receptors containing NR2B subunits. Zn^2+^ elicited a selective increase in NMDA/NR2B fEPSPs, and removal of endogenous Zn^2+^ with high-affinity Zn^2+^ chelators robustly reduced the magnitude of stimulus-evoked LTP. Taken together, our data show that Zn^2+^ at physiological concentrations enhances activation of NMDA receptors containing NR2B subunits, and that this effect enhances the magnitude of LTP.

## Introduction

Plasticity at hippocampal synapses is influenced by a number of endogenous factors including Zn^2+^, which acts on N-methyl-D-aspartate glutamate receptors (NMDAR) involved in the induction of long-term potentiation (LTP). Under normal physiological conditions, Zn^2+^ is released from presynaptic vesicles by low frequency synaptic transmission and interacts with multiple receptors that are involved in the induction of LTP, including NMDAR [1–4].

Studies suggest that Zn^2+^ exerts tonic regulation of NR2A containing NMDARs, and that Zn^2+^ released from presynaptic terminals can also provide activity-dependent phasic modulation/inhibition of NR2B-containing NMDARs [5–7]. Interestingly, removal of vesicular Zn^2+^ via either genetic ablation of the ZnT3 Zn^2+^ synaptic vesicle transporter, or Zn^2+^ chelation with CaEDTA, have been reported to impair LTP, suggesting that endogenous synaptically-released Zn^2+^ may play a role in gating the induction of LTP [8,9]. Izumi and colleagues suggest that removal of Zn^2+^ using CaEDTA, a membrane impermeable metal chelator with high affinity for Zn^2+^ (K_d_ ≈ 10^−16^ M), inhibits LTP due to removal of Zn^2+^ tonically bound to NMDARs [8,10].

Studies suggest that Zn^2+^ released at SCH axons synapsing onto CA1 pyramidal neurons (SCH-CA1) can both inhibit and enhance NMDAR activity [11,12]. The biphasic actions of Zn^2+^ on NMDAR activity likely reflects a combination of free Zn^2+^ concentrations, duration of action, sites of action, and tonic versus phasic properties. Implicated in the mechanisms of possible enhancement vs inhibition are the Src family of tyrosine kinases (SFKs), which are expressed throughout the CNS and are involved in many cellular functions, including regulating ion channel activity (e.g. NMDARs) and synaptic transmission [2, 11–14]. Evidence suggests that Zn^2+^ increases NMDAR responses by increasing SFK activity, which potentiates NMDAR-gated currents [11,15]. SFKs can increase NMDAR-gated currents via phosphorylation of one or more tyrosine residues in NR2A or NR2B subunits [13]. Though several tyrosine residues have been shown to be phosphorylated, the identity of residues that are responsible for increases in NMDAR gating remains unknown [13]. We hypothesized that Zn^2+^ enhances stimulus-evoked LTP through activation of NMDARs containing NR2B subunits activating an SFK-dependent pathway, a hypothesis we tested here at SCH-CA1 synapses in ex vivo mouse hippocampal slices.

## Materials and methods

All experiments were conducted under an approved protocol from the Institutional Animal Care and Use Committee of New York Medical College, in compliance with National Institute of Health guidelines.

### Hippocampal slice preparation

C57/B16 mice (2–3 month old, male and female; Taconic Farms) were deeply anesthetized with isoflurane and decapitated. The brain was removed rapidly, submerged in ice-cold artificial cerebrospinal fluid (ACSF, 2–4°C), which contained: 124mM NaCl, 4mM KCl, 2mM MgSO_4_, 2mM CaCl_2_, 1.25mM NaH2PO_4_, 26mM NaHCO_3_, 10mM glucose; at pH 7.4, and oxygenated continuously with 95% O_2_ and 5% CO_2_. The brain was hemisected through the midsagittal plane, the frontal lobes removed, and each hemisphere glued using cyanoacrylate adhesive to a stage immersed in ice-cold ACSF gassed continuously with 95% O_2_/5% CO_2_ during slicing. 300 μm thick coronal slices were cut using a vibratome (DSK DTK-1000), and transferred to an interface holding chamber containing ACSF with oxygen for incubation at room temperature for a minimum of one hour before commencing recording.

### Extracellular recordings

Brain slices were transferred to an interface recording chamber and continuously perfused at 3 ml/min with oxygenated ACSF at 32 ± 0.5°C. Low resistance recording electrodes were made from thin-walled borosilicate glass (1–2 MΩ after filling with ACSF) and inserted into the apical dendritic region of the SCH field in stratum radiatum of the CA1 region to record evoked field excitatory postsynaptic potentials (fEPSPs). A bipolar stainless steel stimulating electrode (FHC Co.) was placed on SCH-commissural fibers in CA3 stratum radiatum, and constant current stimulus intensity adjusted to evoke approximately half-maximal fEPSPs once each 30 sec (50–100 pA; 100 μs duration). fEPSP slopes were measured by linear interpolation from 20–80% of maximum negative deflection, and slopes confirmed to be stable to within ± 10% for at least 10 min before commencing an experiment.

For all slices, single shock evoked fEPSPs were acquired every 30 seconds, normalized to fEPSP slope amplitude for the 3 minute period immediately prior to application of high frequency stimulation (theta burst stimulation, TBS), and LTP measured as the ratio of mean slope 48–52 minutes post-TBS to the pre-TBS baseline slope. Signals were recorded using a Multiclamp 700B amplifier and digitized with a Digidata 1322 (Axon Instruments, Foster City, CA), and analyzed using pClamp software (version 9, Axon Instruments).

During electrophysiological recordings 1μM ZnCl_2_ was bath applied 15 minutes prior to delivering a TBS in experiments (solid line under the graphs indicates timing of application; arrow indicates TBS), and the slices were perfused continuously for 60 minutes post TBS. The sample traces next to all graphs are averages over 5 minutes (10 traces, 1/30sec) taken from 0–5 minutes prior to TBS (solid trace) and 48–52 minutes post TBS (dashed trace). All baselines for LTP experiments were stable for at least 15 minutes prior to bath application of ZnCl_2_. For all slices, LTP was statistically analyzed 50 minutes post TBS. For experiments examining the effects of baseline to prolonged exposure to ZnCl_2_ without TBS, the magnitude of baseline responses was averaged over 5 minutes during the 15-minute pre-ZnCl_2_ baseline, and compared to the 5 minute average after a 50 minute application of ZnCl_2_.

Monosynaptic SCH-evoked NMDA receptor fEPSP amplitudes were enhanced and isolated pharmacologically by perfusing slices in Mg^2+^-free ACSF, which also contained the AMPA/Kainate receptor antagonist DNQX (25μM). Experiments recording NMDA fEPSPs otherwise followed experimental design as described above.

### Whole-cell voltage-clamp recordings

Patch pipettes were pulled from borosilicate glass (1B150F-4, World Precision Instruments) using a flaming/brown micropipette puller (P-97, Sutter Instruments). The composition of the patch pipette solution for NMDA current recordings was: 135mM CsMeSO_3_, 8mM NaCl, 10mM HEPES, 2mM Mg-ATP, 0.3mM Na-GTP, 0.5mM EGTA, 1mM QX-314. The patch pipette solution pH was adjusted to 7.25 with CsOH, and had an osmolarity of 280 ± 10 mOsm. When filled with this solution, patch pipettes had tip resistances of 5–6 MΩ. Whole cell patch clamp recordings were obtained from CA1 pyramidal neurons in slices in a fully submerged recording chamber at room temperature, perfused with ACSF at 2 ml/min, and gassed continuously with 95% O_2_/5% CO_2,_ which passed over the perfusate and bubbled in ACSF.

NMDA evoked currents were pharmacologically isolated by bath application of tetrodotoxin (TTX) to eliminate spontaneous action potential driven synaptic release, plus the GABA receptor antagonist bicuculline (25μM), AMPA receptor blocker DNQX (25μM), and intracellular perfusion with the K+ channel blocker (Cs^+^, 135mM) in pyramidal neurons voltage clamped at -70mV. A glass pipette was placed 150–200 micrometers from the pyramidal cell body in SCH synapses in stratum radiatum, and puffing pressure adjusted to elicit approximately 100pA amplitude inward currents. NMDA puffed every 90 seconds was preferred to allow the NMDA receptor to recover and avoid desensitization, and a 10 minute stable baseline of NMDA evoked currents confirmed before proceeding with the experiment. NMDA evoked current traces were averaged (4 traces) at 5 minutes and at 40 minutes (or after 40 minutes in ZnCl_2)_ recorded in Mg^2+^-free ACSF also containing bicuculline (25μM) plus DNQX (25 μM).

Paired-pulse facilitation (PPF) experiments measure the amplitude difference in AMPA excitatory postsynaptic current (EPSC) at increasing time intervals. PPF given at the intervals of 10, 20, 50, 100, 250, and 500 ms, recorded after 15 minutes exposure to ZnCl_2._ PPF traces demonstrate the difference from initial simulation and after 50 ms. The horizontal lines on the traces indicating the peak amplitude along with the vertical double-headed arrow demonstrates the relative increase in AMPA EPSC amplitudes of the paired-pulse facilitation. The mean PPF ratios of AMPA EPSC amplitudes were calculated using the second stimulus divided by the first stimulus.

The submerged recording chamber was mounted on a Zeiss Axioskop 2 FS upright microscope equipped with infrared differential interference contrast (DIC) optics. Pyramidal neurons of the hippocampal CA1 region were visualized with a 63x water immersion lens, and patched in the voltage-clamp configuration. NMDAR evoked currents and AMPA EPSCs were recorded using a MultiClamp 700B (Molecular Devices, Foster City, CA), with the low-pass filter setting at 1–3 kHz, series resistance was compensated in the voltage-clamp mode, and patched cells whose series resistance changed by more than 10% were rejected from further analysis. Data were acquired with a 16-bit D/A interface (Digidata 1322A, Molecular Devices) stored on a PC-compatible computer and analyzed using PCLAMP software (v9, Molecular Devices).

### Chemicals

All external and patch pipette solutions were made with deionized distilled water (Milli-Q system). The chemicals for making extra- and intracellular solutions were purchased from Sigma (St Louis, MO) and Fluka (New York, NY). Neurotransmitter receptor antagonists were purchased from Tocris (Ellisville, MO).

### Western blot analyses

Western blotting was performed on mouse hemi-sectioned brain slices (300 μm thick), which included not only the hippocampus but overlying cortex as well. Slices were treated in pairs as control (ACSF alone), control + Zn^2+^; Zn^2+^ + PP2; control + PP2. Each condition was tested on every animal using multiple slices. Each animal provided 2 control slices, 2 Zn^2+^ slices, one slice treated with PP2 with Zn^2+^ and one slice with PP2 alone. (The PP2 alone treatment was not different from control–data not shown.) The control group was exposed to ACSF for 35 minutes, while the treated groups received 10-minute exposure to ACSF, followed by 30 minute exposure to ZnCl_2_ (1μM) or 30 minute exposure to 1 μM ZnCl2 in the presence of the tryrosine kinase (SFK) inhibitor 1-(1,1-dimethylethyl)-1H-pyrazolo[3,4-d]pyrimidin-4-amine (PP2, 10μM). Slices were then transferred to microcentrifuge tubes and flash frozen with liquid nitrogen and stored in a -80°C freezer.

Frozen tissue samples were boiled for 5 minutes and homogenized with high speed vortexing in lysis buffer (4% LiDS, 10% glycerol, 62.5 mM of TRIS-HCl pH 6.8, and 10 mM DTT). The samples were centrifuged afterwards for 5 minutes and the protein concentration was determined using a protein assay kit (Bio-Rad). 15 μg of protein was separated on 4–15% SDS-PAGE gradient precast gel (Bio-Rad). A separate gel was run for each animal. For each gel, equal aliquots were run in duplicate for each slice in adjacent lanes. This pattern was duplicated on the same gel so that all samples from the same animal were transferred to the PVDF membrane at the same time under the same conditions. After electroblotting, the membrane was cut in half and one half was developed with anti NMDAR2B antibody to visualize the total amount of receptor present in the sample and the other half was developed with anti PY (TYR1472) NMDAR2B antibody. Membranes were blocked with casein then incubated with primary antibodies at room temperature overnight followed by 1hr washing in TBST (0.05% Tween 20, Tris buffered saline) at RT. Primary antibodies were NMDAR/NR2B and phospho-NR2B Y1472 primary antibodies (Cell Signaling #4207 and 4208) and anti-β-actin to normalize band intensities. Secondary antibodies conjugated with horseradish peroxidase (Jackson ImmunoResearch) were visualized using chemiluminescence ECL-Plus reagent (GE Healthcare).

The blots were densitometrically analyzed using Image-J. Quantitative comparisons were performed by normalizing each blot to a control lane and the relative value of the PY signal was divided by the total NMDAR2B signal. Thus a ratio of one is the value of control standard- the PY/NMDA for control is set to 1.

### Data analysis

Electrophysiological data were analyzed initially with Clampfit (v9) (Axon Instruments, CA). Analyzed data were further processed and presented with Origin 6.1 (Microcal Software, Northampton, MA) and CorelDraw 10.0 (Corel, Ottawa, Ontario, Canada) programs. Statistical analysis were performed with SPSS (v11). Statistical data are presented as mean ± SEM unless indicated otherwise. Significance level was preset to *P*<0.05. Data points averaged for statistics are marked with a dashed bracket over the graphs. All data points analyzed were a 5 minute average per slice from the time points indicated by the brackets and are taken from within the last ten minutes of recording. Means ± SEM were calculated for each group and then compared to other experimental groups using 1-way ANOVA and Dunnett’s multiple comparison test. The number of slices used for analysis included comparison of slices from within the same mice and between slices of different mice.

## Results

### I. Zinc enhances stimulus-evoked LTP at Schaffer collateral-CA1 synapses

**Fig 1:**
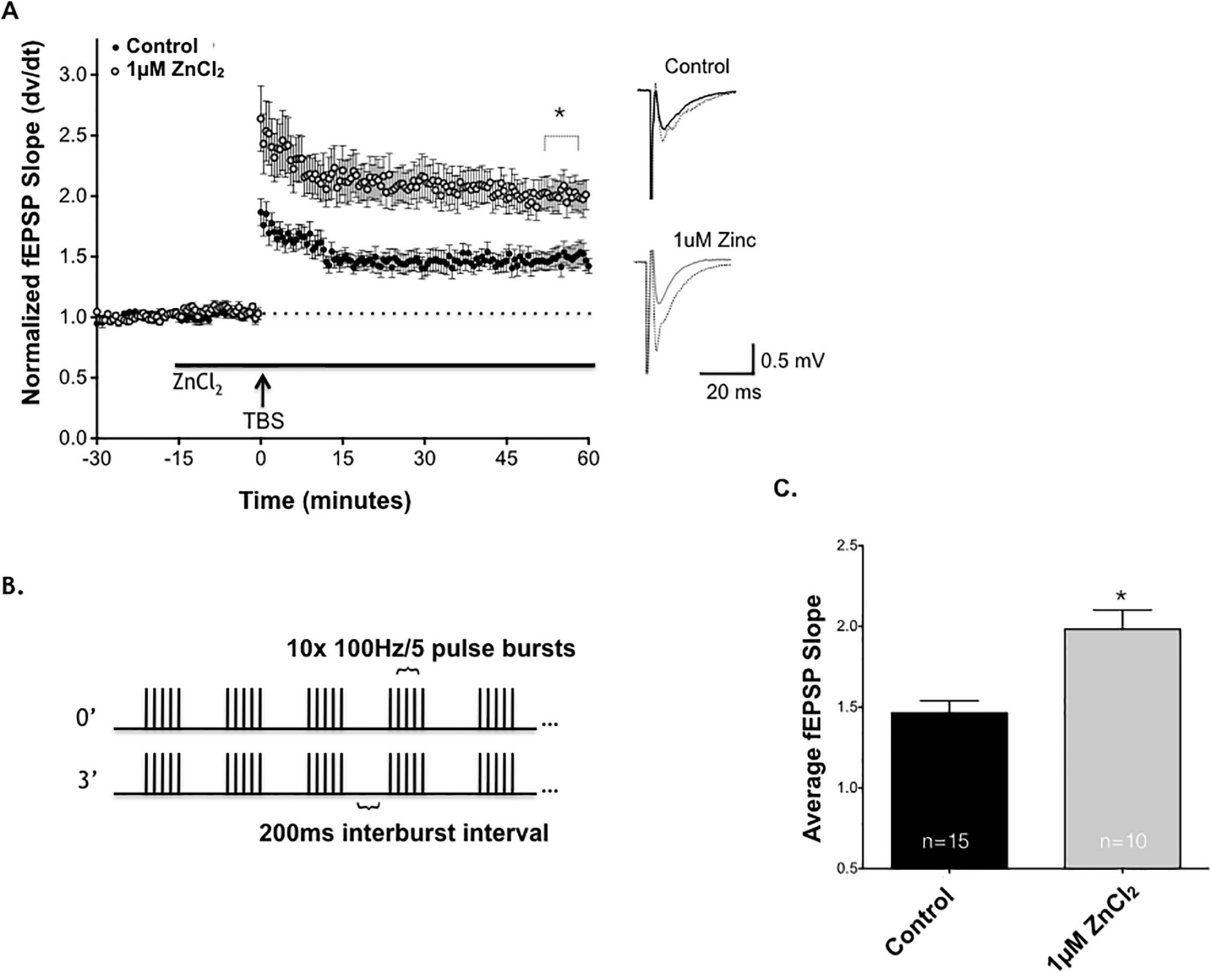
1μM ZnCl_2_ enhances the magnitude of long-term potentiation (LTP) at Schaffer collateral-CA1 synapses in hippocampus. **(A)** Time course of TBS-induced LTP of SCH-CA1 fEPSP slopes in control (dark circles, n = 15), compared to 1μM ZnCl_2_ (lighter circles, n = 10). Bath-applied 1μM ZnCl_2_ (solid bar) significantly and persistently enhanced magnitude of LTP compared to untreated control slices. LTP was significantly enhanced 50 minutes post TBS (*, P<0.05; Student’s t-test). Sample traces next to graph compare baseline (solid wave) to post TBS (dashed wave) for all conditions. **(B)** The theta burst stimulus (TBS) stimulating protocol used for all LTP experiments (2 trains 3 minutes apart, each train consisting of 10x 100Hz/5pulse bursts at 200ms interburst intervals).**(C)** Summary of all control slice LTP vs ZnCl_2_ treated slices, demonstrating that ZnCl_2_ significantly enhances SCH-CA1 LTP at 1μM (*, P<0.05; Student’s t-test). Statistical data are presented as mean ± SEM.

To test the potential role of Zn^2+^ in regulating synaptic plasticity, we commenced by bath applying exogenous Zn^2+^ to mouse hippocampal slices at a concentration of 1μM to evaluate stimulus-evoked LTP at SCH synapses in field CA1. Fig 1A illustrates SCH-evoked field excitatory postsynaptic potentials (fEPSPs) recorded from stratum radiatum of field CA1 before and after a high frequency stimulus was applied to SCH axons using a theta-burst protocol (Fig 1B).

ZnCl_2_ (1μM) did not significantly alter fEPSP slope during the 15 minutes of baseline recording prior to high frequency stimulation (TBS). However 1μM ZnCl_2_ significantly increased the magnitude of LTP after TBS. Bath application of ZnCl_2_ at concentrations of 0.01μM and 10μM, did not significantly enhance the magnitude of LTP after TBS stimulation (S1 Fig). Fig 1C shows the average fEPSP response at 50 minutes post TBS in control vs 1μM ZnCl_2._ 1μM ZnCl_2_ has been posited to be the physiological concentration of actively released Zn^2+^ within SCH-CA1 synapses [16]. Zn^2+^ has been shown to inhibit recombinant NMDA receptor-gated conductance at concentrations ranging from 1–100μM, which would be consistent with an inhibitory role in LTP [17]. Moreover, 1–5μM Zn^2+^ is thought to be sufficient to bind to the low affinity, allosteric inhibitory site on NR2B containing NMDA receptors [17]. Our data suggests there is an optimal concentration of Zn^2+^ that can potentiate SCH-CA1 LTP.

### II. Chelation of endogenous zinc inhibits LTP at Schaffer collateral-CA1 synapses in hippocampal slices

While the previous experiment addressed the mechanisms of modulation of synaptic transmission and LTP by physiological concentrations of exogenously-applied Zn^2+^ (1μM), it remained to be determined whether endogenous Zn^2+^ released by SCH terminals can influence LTP in a similar manner. The next set of experiments were designed to determine the effects of chelating endogenous Zn^2+^ on LTP, using the membrane impermeable Zn^2+^ chelator CaEDTA [8,18], or the cell permeable Zn^2+^ chelator N,N,N’,N’-Tetrakis (2-pyridylmethyl) ethylenediamine (TPEN).

**Fig 2:**
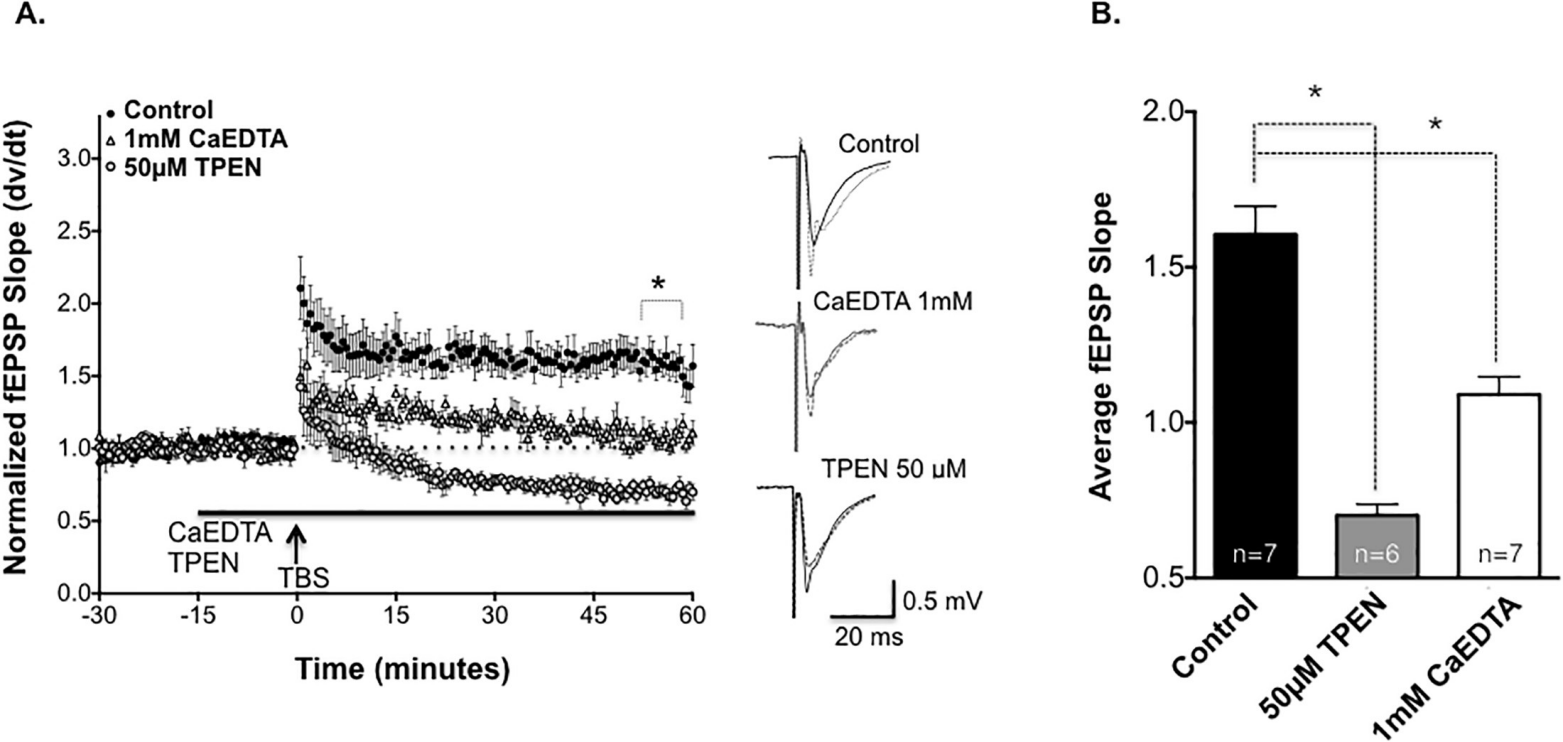
Chelation of endogenous Zn^2+^ inhibits CA1-LTP. **(A)** Bath application (solid bar) of the Zn^2+^ chelater 1mM CaEDTA (open triangles, n = 7) significantly reduced the magnitude of SCH-CA1 LTP compared to untreated control slices (dark circles, n = 7). Bath application of the Zn^2+^ chelator 50μM TPEN (solid bar) converted control LTP to LTD (light circles, n = 6; Comparison of three groups *P*<0.05; 1-way ANOVA). Sample traces next to graph compare baseline (solid wave) to post TBS (dashed wave) for all conditions. **(B)** Summary of mean ± SEM fEPSP slopes 50 minutes post-TBS for untreated control slices versus slices pre-treated with 1mM CaEDTA (*, *P*<0.05; Dunnett’s post hoc test) and slices pre-treated with 50μM TPEN, where LTP converted to LTD (*, *P*<0.05; Dunnett’s post hoc test).

CaEDTA (1mM) significantly inhibited TBS-induced LTP (Fig 2A). Fig 2B shows the average fEPSP response to chelation of Zn^2+^. It is important to note that the kinetics of CaEDTA binding to Zn^2+^ is likely too slow (60 milliseconds) to bind to synaptically released Zn^2+^, because CaEDTA must first dissociate Ca^2+^ ions before binding to Zn^2+^ [19]. Therefore, CaEDTA was used to assess the relative importance of *tonic* extracellular Zn^2+^ on SCH-CA1 LTP.

We next tested the effects on LTP of a cell-permeant chelator, TPEN, with a higher affinity for Zn^2+^ than CaEDTA. TPEN has a high affinity for Zn^2+^ and low affinity for Ca^2+^ (Zn^2+^ K_d =_ 10^−16^ M vs Ca^2+^ K_d =_ 10^−5^ M) and Mg^2+^ (Kd = 10^-2^M) [20, 21]. TPEN (50μM) did not alter baseline fEPSPs, suggesting no alteration in basal AMPAR-mediated low-frequency synaptic transmission or non-specific synaptotoxicity. In sharp contrast to control slice LTP, TPEN converted the effects of TBS to a long-term depression (LTD) of synaptic transmission. This confirms that Zn^2+^ is necessary for full expression of SCH-CA1 LTP, but not LTD, and suggests that both extracellular and intracellular Zn^2+^ are required. It should be noted that CaEDTA and TPEN not only differ in their kinetics, but have differing sites of action (solely extracellular vs extracellular plus intracellular), making the ultimate site(s) of action of Zn^2+^ unclear.

### III. Zinc enhancement of Schaffer Collateral-CA1 LTP requires postsynaptic NMDA receptor activation

**Fig 3:**
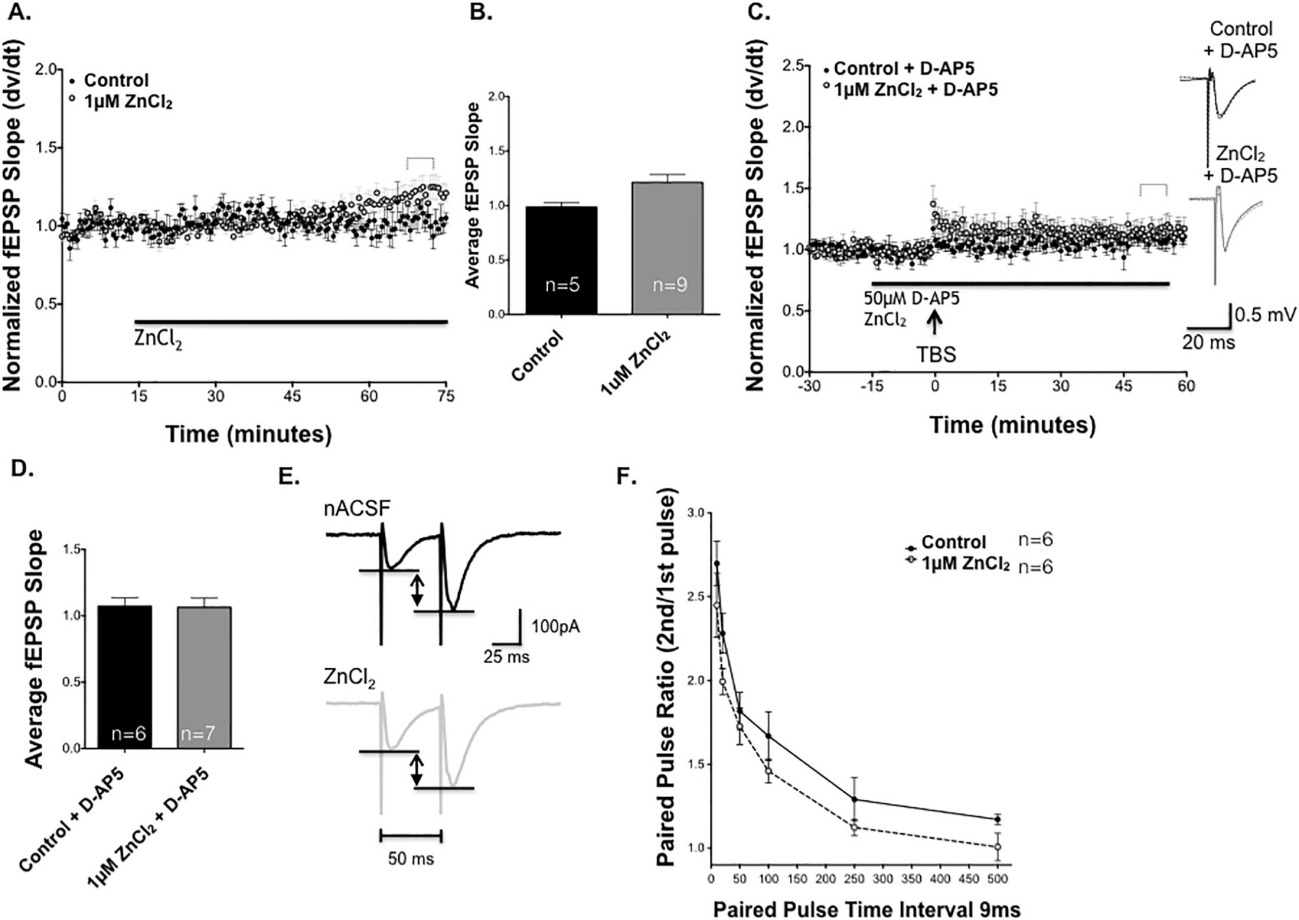
Zn^2+^ enhancement of LTP requires activation of postsynaptic NMDA receptors. **(A)** ZnCl_2_ (1μM, light circles, n = 9) does not alter basal synaptic transmission after 1 hour of exposure compared to control slices (dark circles, n = 5; *P*>0.05, Student’s t-test). **(B)** Summary of baseline responses showing that ZnCl_2_ did not alter baseline synaptic transmission compared to untreated control slices. **(C)** TBS did not induce LTP in the presence of either 50μM D-AP5 (filled circles, n = 6), or 1μM ZnCl_2_ + 50μM D-AP5 (light circles, n = 7). **(D)** Summary of mean fEPSP slopes 50 minutes post TBS. There was no significant difference between D-AP5 alone and 1μM ZnCl_2_ LTP + D-AP5 at 50 minutes post TBS. (*P*>0.05, Student’s t-test) **(E)** Sample of control paired-pulse evoked SCH-CA1 EPSCs (dark trace, average of 6 responses) versus EPSCs in 1μM [ZnCl_2_] (light trace, average of 6 responses) during a paired-pulse stimulus at an interval of 50ms. **(F)** Paired-pulse profiles of PPF ratios at interpulse intervals from 10–500 msec in control (n = 6, dark circles) vs. 1μM [ZnCl_2_] (n = 6, gray circles), showing no significant differences (*P*>0.05, Student’s t-test). Statistical data are presented as mean ± SEM.

While the previous experiments did not reveal any acute baseline effects of Zn^2+^ application on SCH-CA1 evoked synaptic transmission over a 15 minute application, we further examined the effects of Zn^2+^ on basal synaptic transmission for an extended period up to one hour (Fig 3A and 3B). SCH-CA1 fEPSP slopes were evoked once every 30 seconds for up to one hour without TBS. In the presence of 1μM ZnCl_2_, baseline fEPSPs did not show a change in basal synaptic activity after 50 minutes in ZnCl_2_, indicating that low-frequency synaptic transmission was not affected by Zn^2+^.

To test whether NMDA receptor activity was necessary for Zn^2+^-induced enhancement of LTP, we tested whether the effect could be blocked by the selective NMDAR antagonist 2-amino-5-phosphonopentanoic acid (D-AP5). In control slices, 50 μM D-AP5 was sufficient to completely block NMDA receptors and TBS-induced LTP (Fig 3C). Moreover, the addition of 1μM ZnCl_2_ in the presence of D-AP5 was unable to rescue LTP (Fig 3C). Fig 3D compares the average fEPSP of control versus Zn^2+^ and D-AP5 at 50 minutes post TBS. These experiments demonstrate that SCH-CA1 LTP elicited by our TBS stimulus protocol is an NMDAR-mediated phenomenon, and that the enhancement of LTP produced by Zn^2+^ does not appear to be via an enhancement of non-NMDAR mediated LTP.

While the previous experiment suggests NMDA receptors play a key role in the enhancement seen by ZnCl_2_, it is unclear whether the enhancement of LTP was due to effects on presynaptic release of glutamate, and/or postsynaptic responsiveness of NMDA receptors to glutamate. To test whether Zn^2+^ altered presynaptic transmitter release probability, we used whole-cell voltage-clamp recording to isolate AMPAR excitatory postsynaptic currents (EPSCs) for measurement of paired-pulse facilitation (PPF). The differences in EPSCs magnitude between groups were measured as demonstrated in Fig 3E. PPF was not significantly different in the presence and absence of 1μM ZnCl_2_ (50 ms interval; Fig 3E and 3F). There was no significant difference in PPF ratio at any of the time intervals tested (Fig 3F), or in basal synaptic EPSCs, produced by 1μM ZnCl compared to untreated control slices, suggesting that this concentration of Zn^2+^ does not alter SCH presynaptic release probability or CA1 pyramidal cell postsynaptic AMPAR-mediated EPSCs.

### IV. Zn^2+^ enhances NMDA receptor fEPSPs and whole-cell pharmacologically-isolated NMDA receptor evoked currents in CA1 pyramidal neurons

An increase in NMDAR activity associated with both increases in channel current and channel number have been correlated with enhanced magnitude of LTP [22, 23]. Zn^2+^, acting either directly or indirectly on NMDAR-gated conductance, is a reasonable hypothetical mechanism for its enhancement of LTP. Since D-AP5 blocked Zn^2+^ enhancement of NMDAR-dependent LTP, we postulated that Zn^2+^ would increase NMDAR-mediated fEPSPs at 1μM ZnCl_2_. In these experiments, fEPSP amplitudes were measured instead of slopes because of the significantly different activation and deactivation kinetics of NMDA receptor subunit types [24–27], and the fact that these synapses contain a mixture of NMDA receptor subtypes, making slope inaccurate as a reflection of the overall change in all NMDA receptor responses. For example, a significant increase in the proportion, total number, or a change in the state of phosphorylation of NR2B mediated responses could all produce an increase in amplitude, but a decrease in slope, due to its slower activation kinetics [24–27].

**Fig 4:**
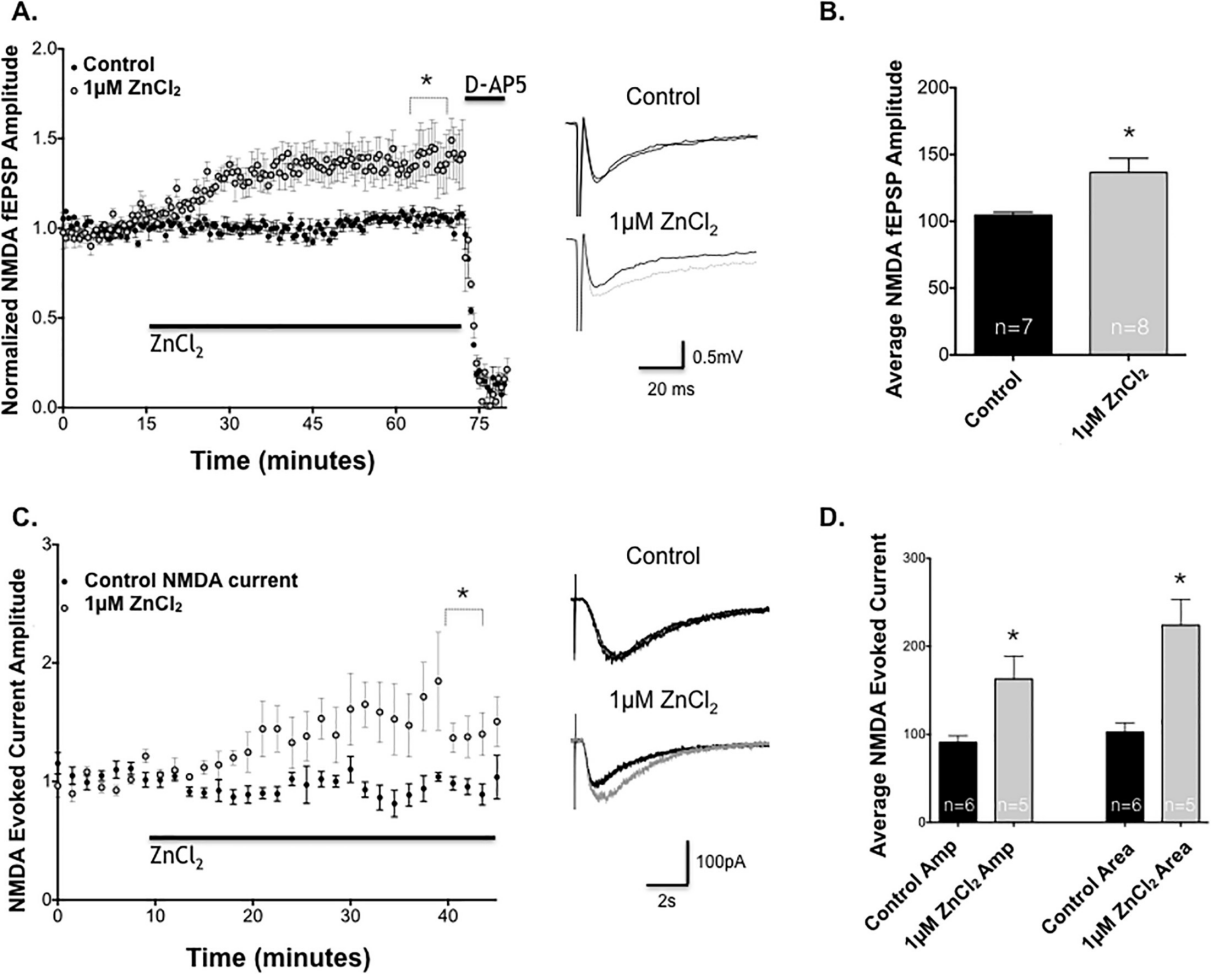
ZnCl_2_ enhances NMDA receptor-dependent fEPSP and NMDA evoked currents. **(A)** Effect of bath application of ZnCl_2_ on pharmacologically-isolated NMDAR fEPSPs. ZnCl_2_ (black bar) produced a slow, persistent enhancement of NMDAR fEPSPs (light circles, n = 7) compared to untreated control slices (dark circles, n = 8). D-AP5 (50μM) was bath applied after one hour (n = 2 for control and ZnCl_2_), which eliminated NMDAR fEPSP responses in both groups. **(B)** Summary of mean ± SEM NMDAR fEPSP amplitude % increase over baseline after 1 hour bath application of ZnCl_2_ in control vs 1μM ZnCl_2_ (*, *P*<0.05; Student’s t-test). **(C)** Effect of bath application of 1μM ZnCl_2_ (light circles, n = 5) on pharmacologically-isolated SCH NMDA evoked currents in CA1 pyramidal neurons by pressure injection of NMDA compared to untreated control neurons (dark circles, n = 6). 1μM ZnCl_2_ significantly and persistently increased NMDA evoked currents amplitudes in CA1 pyramidal neurons. **(D)** Mean ± SEM NMDA evoked currents amplitude (*; *P*<0.05, Student’s t-test) and total area (*; *P*<0.05, Student’s t-test), calculated as the % increase over baseline after 40 minutes ZnCl_2_ application, compared to its control baseline.

Consistent with this hypothesis, 1μM ZnCl_2_ significantly enhanced synaptically-evoked pharmacologically-isolated NMDAR fEPSPs (see Methods) in CA1 pyramidal neurons (Fig 4A and 4B), and this effect was eliminated by addition of D-AP5 after the enhancement of ZnCl_2_ was established, confirming that the effect of Zn^2+^ was NMDA receptor mediated. In contrast, neither 0.01 nor 10 μM Zn^2+^ significantly altered NMDAR-mediated fEPSPs (S2A Fig). Moreover, the enhanced effect after application of 1 μM ZnCl_2_ on NMDAR fEPSPs was absent in solutions containing magnesium (S2B Fig), suggesting that Zn^2+^ requires the removal of the Mg^2+^ NMDA receptor block in order to exert its effect as would occur during high frequency stimulation to induce LTP.

Having found that low micromolar concentrations of Zn^2+^ enhances NMDAR-mediated synaptic transmission, we next examined whether this action was due to a direct effect on postsynaptic NMDA-mediated currents. Whole-cell patch clamp with local puff application of NMDA was used to further isolate NMDA receptor responses. Within 10–15 minutes of bath application of 1μM [ZnCl_2_], NMDA evoked current amplitude increased significantly compared to untreated control CA1 pyramidal neurons (Fig 4C and 4D).

In addition to an increase in NMDA evoked current amplitude, cells treated with 1μM Zn^2+^ demonstrated a significant increase in the area under the evoked current waveform, indicative of an increase in charge transfer (S3 Fig). Fig 4D compares evoked current amplitude and area under the curve to control NMDA evoked currents. NMDAR channels are nonselective cation channels, and an increase in charge transfer across the neuronal membrane would likely include an increase in postsynaptic Ca^2+^ influx, a second messenger necessary to trigger the induction of LTP. This increase in area suggests either greater activation, increase in total receptor number, or a change in the state of conductance of NR2B-containing NMDARs, which have slower deactivation kinetics than NR2A-containing NMDAR [28]. It is important to note that the concentration of NMDA delivered by pressure ejection could not be precisely measured, but is likely sufficient to activate both synaptic (predominantly NR2A-containing) and extrasynaptic (mostly NR2B-containing) NMDAR. Taken together, these results suggest that 1μM ZnCl_2_ is capable of increasing NMDA current amplitude and evoked current area, indicative of increased charge transfer that may be due to NMDA subunit-specific NMDAR modulation. The next experiments were designed to test the hypothesis that Zn^2+^ exerts its increase on NMDA receptor current via interaction with NR2B-containing NMDARs.

### V. Zn^2+^ enhancement of NMDA fEPSPs is blocked by inhibition of NR2B-containing NMDAR, and of Src family tyrosine kinases

**Fig 5:**
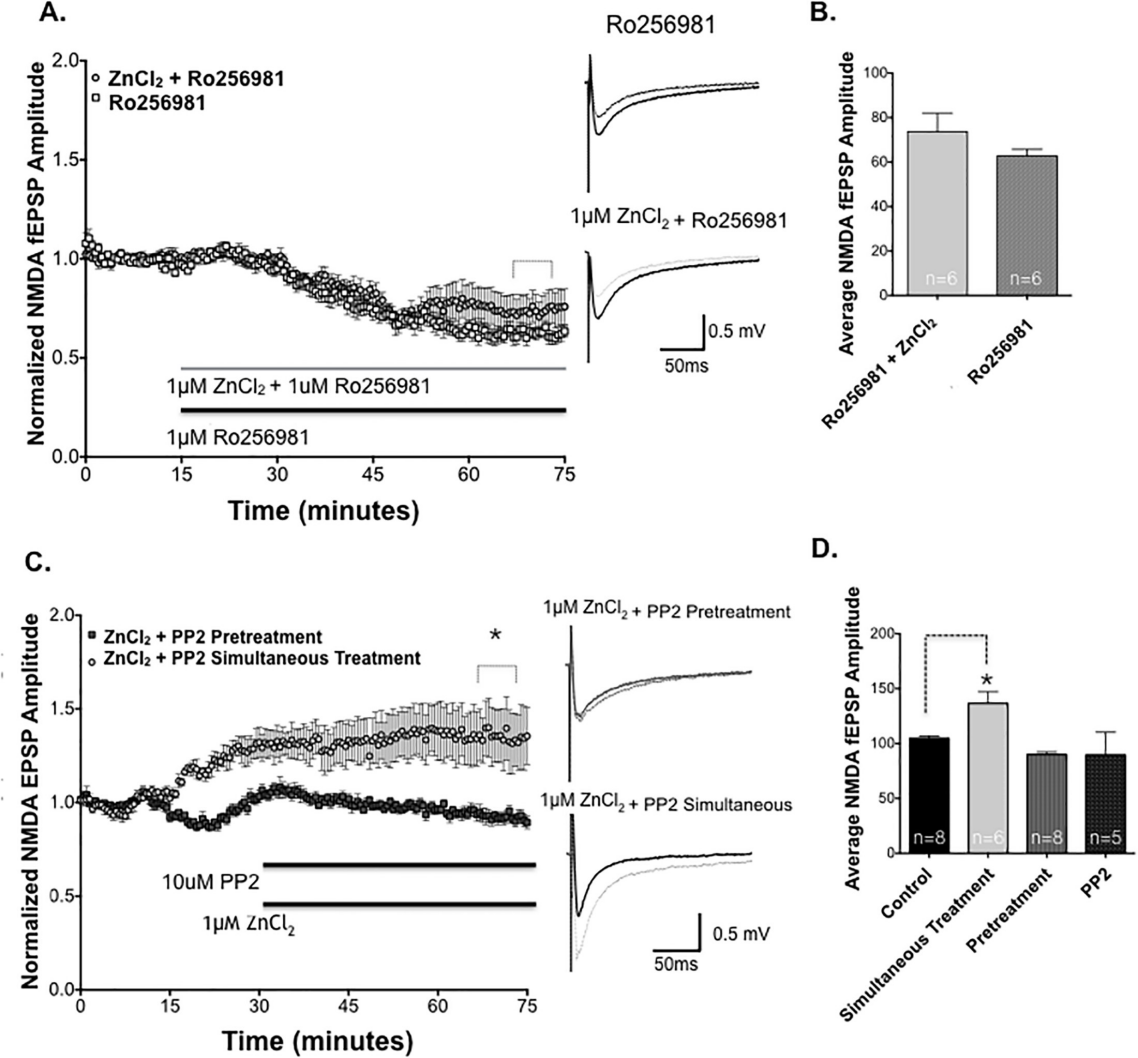
Zn^2+^ is required for the enhancement of NMDAR fEPSPS which requires NR2B-containing NMDARs and activation of Src family kinases (SFK). **(A)** Pharmacologically-isolated NMDA fEPSP amplitudes in the presence of the NR2B-selective NMDAR antagonist Ro25-6981 (1 μM) in control slices (squares, n = 6), and after co-application of 1μM ZnCl_2_ (light circles, n = 6). Ro25-6981 completely blocked the enhancement of NMDAR fEPSPs by 1μM ZnCl_2_. Sample traces for each treatment shown to right, before (dark traces) and 50 minutes after (light traces) drug application. **(B)** Summary of NMDAR fEPSP decrease versus baseline (100%) after 70 minutes drug application in slices treated with 1μM Ro25-6981 and those treated with Ro25-6981 plus 1μM ZnCl_2_ (*P*>0.05; Student’s t-test). **(C)** Time course of NMDA fEPSP amplitudes in slices pre-treated for 5 minutes with the SFK inhibitor PP2 (top bar, 10μM, dark squares, n = 8) prior to bath application of 1μM ZnCl_2_ (lower bar) plus PP2, versus application of the two drugs simultaneously (light circles, n = 6). **(D)** Summary of NMDA fEPSP amplitudes after 1 hour recording. Simultaneous treatment with 1μM ZnCl_2_ + PP2 significantly enhanced the magnitude of NMDA fEPSPs compared to control ACSF (*, *P*<0.05; 1-way ANOVA, Dunnett’s multiple comparison test), and this potentiation was completely blocked by pretreatment with 10μM PP2 (Control n = 8, vs pretreatment, P>0.05; Dunnett’s multiple comparison test). Mean NMDA fEPSPs in PP2 alone was not statistically different from control (Control vs PP2, P>0.05; Dunnett’s multiple comparison test). Statistical data are presented as mean ± SEM.

To selectively explore the influence of Zn^2+^ on NR2B-containing NMDARs, we utilized the activity-dependent NR2B-selective NMDAR antagonist Ro25-6981 [29], to test the effect of Zn^2+^ on pharmacologically isolated NR2A-containing NMDAR fEPSPs (Fig 5A). Ro25-6981 was used at a concentration that maximally inhibits NR2B-NMDARs, while avoiding inhibition of NR2A-NMDARS (1μM; NR2B IC_50_ ≈ 5-50nM, NR2A IC_50_ ≈ 50μM, 30,31). Ro25-6981 reduced NMDA fEPSP amplitude below baseline (*, *P*<0.05 Student’s t-test, 35.3% reduction compared to baseline), suggesting a population of NR2B-activated NMDA receptors are present in CA1 synapses. When 1μM Ro25-6981 and 1μM ZnCl_2_ were added together to the perfusate, NMDAR fEPSPs were reduced in a fashion nearly identical to Ro25-6981 alone (Fig 5B). These data indicate that targeted blockade of NR2B-NMDARs completely blocks the effects of Zn^2+^ on SCH-evoked NMDAR fEPSPs.

To confirm the finding that Zn^2+^ enhancement of NMDARs is indeed blocked by NR2B inhibition, we performed a second experiment using ifenprodil, a selective NR2B-containing NMDAR antagonist from which the more potent Ro25-6981 is derived. Ifenprodil also reduced NMDA fEPSPs (S4A Fig). When 1μM ZnCl_2_ was added in the presence of ifenprodil, the enhancement by Zn^2+^ was completely abrogated, consistent with a selective action of Zn^2+^ on NR2B-containing NMDARs.

Co-application of 1μM ZnCl_2_ and 50nM NVP AAM077 failed to enhance NMDAR EPSPs (S4C Fig). (Of note: NVP AAM077 has only modest selectivity for NR2A over NR2B, so the moderately larger reduction in NMDA EPSPs is likely a reflection of antagonism of multiple NMDA receptor subtypes [30, 31]). Taken together, our data strongly suggest that NR2B-containing NMDARs are targets of Zn^2+^ modulation of NMDAR synaptic transmission that regulates LTP amplitude.

To further elucidate the mechanism by which ZnCl_2_ modulates NR2B-containing NMDARs, we next explored the Src family of tyrosine kinases (SFKs), which have been shown to regulate NMDAR activity [32, 33]. SFKs have been shown to potentiate the efficacy of hippocampal mossy fiber CA3 pyramidal synapses through activation of the tyrosine kinase receptor, TrKB [33]. We hypothesized that low micromolar Zn^2+^, either through direct or indirect mechanisms (i.e. TrKB activation), could activate SFKs, and that this could lead to potentiation of NMDAR synaptic transmission [32, 33].

We applied 1μM ZnCl_2_ simultaneously co-perfused with 10μM PP2, which it produced the same enhancement of NMDAR fEPSPs as Zn^2+^ alone (Fig 5C). However, since it was possible that Zn^2+^ traversed the plasma membrane more rapidly than the bulkier PP2 molecule, to act before sufficient inhibition of SFK was achieved, we also bath applied PP2 5 minutes *prior to* the application of 1μM ZnCl_2_ in the experiments shown in Fig 5C. Pretreatment with PP2 5 minutes prior to ZnCl_2_ application completely prevented the enhancement of LTP by 1μM ZnCl_2_, returning it to the level of untreated control slices and PP2-only treated slices (Fig 5C and 5D). These results suggests that Zn^2+^ requires the activation of SFKs to increase NMDAR fEPSPs.

### VI. Zinc enhancement of LTP requires NR2B-containing NMDA receptors and activation of the SFK pathway

**Fig 6:**
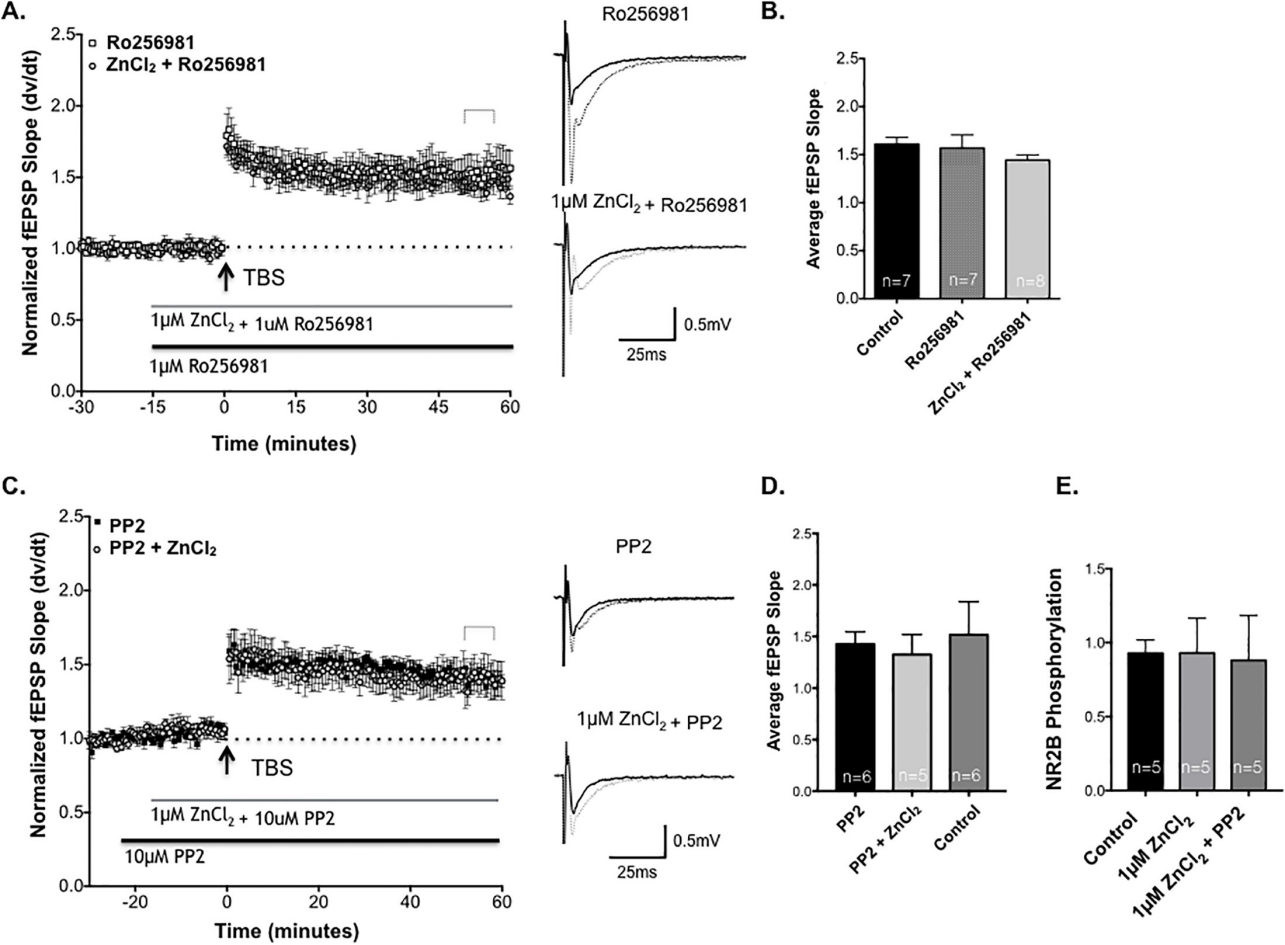
NR2B blockade and Src kinase inhibition block Zn^2+^ enhancement of Schaffer collateral-CA1 LTP. **(A)**Time course of LTP of SCH-evoked fEPSPs in 1μM ZnCl_2_ + Ro 25–6981 slices (circles, n = 8), compared to LTP in slices treated with 1μM Ro 25–6981 (black bar, squares, n = 7). There was no significant difference in the magnitude of LTP between groups. Sample traces for each treatment shown to right, before (dark traces) and 50 minutes after (light traces) TBS. **(B)** Summary of SCH-CA1 LTP 50 minutes post-TBS in slices treated with Ro25-6981 and Ro25-6981 + ZnCl_2_, compared to drug-free control slices (n = 7; *P*>0.05; 1-way ANOVA). **(C)** Time course of LTP elicited by TBS (arrow) in the presence of 10μM PP2-treated slices (squares, n = 6), compared to LTP in PP2 + 1μM ZnCl_2_ treated slices (circles, n = 5). The magnitude of LTP in the two groups were not significantly different (P>0.05; Student’s t-test). Sample traces for each treatment shown to right, before (dark traces) and 50 minutes after (light traces) drug application. **(D)** Summary of normalized fEPSP slope 50 minutes after TBS in slices treated with 1μM ZnCl_2_ + PP2 versus PP2. Statistical data are presented as mean ± SEM. **(E)** Western blot analysis showing the Mean ± SEM of phosphorylation at Y1472 on NMDA NR2B receptor subunits in the untreated control slices, compared to slices treated with 1μM ZnCl_2_ or with 1μM ZnCl_2_ and the PP2 inhibitor. Zn^2+^ treatment did not significantly alter the state of phosphorylation of the NR2B subunits at Y1472 (*P>*0.05; Dunnett’s multiple comparison test).

Next we evaluated whether enhancement of NR2B NMDA receptor activation is necessary for Zn^2+^-induced enhancement of SCH-CA1 synapse LTP. We examined the effects of selectively blocking NR2B-containing NMDARs on stimulus-evoked LTP. We added 1μM Ro25-6981, followed by SCH TBS. Ro25-6981 had a minimal effect on the magnitude of LTP compared to control slices (Fig 6A and 6B). These data are consistent with other reports indicating that inhibition of NR2B-containing NMDARs may not markedly inhibit the induction or expression of LTP under control conditions [34].

Co-application of 1μM ZnCl_2_ + Ro25-6981 completely eliminated Zn^2+^ enhancement of LTP without altering the magnitude of baseline control LTP (Fig 6A and 6B). These data support the hypothesis that Zn^2+^ enhances LTP by selectively modulating activation of NR2B-containing NMDA receptors.

Since SFK inhibition blocked Zn^2+^ enhancement of NMDAR fEPSPs, it was a reasonable hypothesis that PP2 would also specifically prevent Zn^2+^ enhancement of stimulus-evoked LTP at Schaffer-Collateral CA1 synapses. LTP of SCH-evoked fEPSPs in field CA1 in the presence of PP2 (10μM) alone, versus PP2 co-applied with 1μM ZnCl_2_ (Fig 6C and 6D), showed no difference in magnitude of LTP, confirming that SFK activity is necessary for the enhancement of LTP by Zn^2+^.

As an additional control for nonspecific effects of PP2, we compared the effects of the inactive PP2 analog PP3. ZnCl_2_ markedly enhanced the magnitude of TBS-induced LTP in the presence of 10μM PP3 compared to PP2 (S5 Fig). Taken together, these data support the conclusion that the potentiation of LTP by Zn^2+^ requires SFK activity.

Our final objective was to begin to probe the site(s) of phosphorylation of NR2B subunits that may be involved in Zn^2+^ regulation of LTP. Tyrosine 1472 (Y1472) is the main phosphorylated tyrosine residue on the C-terminal tail of the NR2B subunit [13]. Under basal conditions, this site is phosphorylated, and there is an increase in phosphorylation at this site following tetanus-induced LTP in the dentate gyrus [13]. Phosphorylation of this site is correlated with an increase in surface expression of NMDA receptors and a corresponding reduction in the intracellular pool of NMDA receptors [13]. Increased surface expression of NR2B containing NMDARs due to an increase in Y1472 phosphorylation could account for an increase in NR2B current after low micromolar ZnCl_2_ application. Our next experiment examined whether exposure to Zn^2+^ altered baseline NMDA receptor phosphorylation at Tyrosine 1472.

Western blots were probed with an antibody to the phosphorylated site Y1472 on the NR2B subunit to test this hypothesis (S6 Fig). ZnCl_2_-treated (1μM) slices exhibited no significant change in phosphorylation compared to baseline ACSF controls slices (Fig 6E). Therefore, simply exposing slices to Zn^2+^ is not sufficient to alter phosphorylation of the NR2B subunit at the Y1472, suggesting that other phosphorylation sites may be involved or that it may require the NMDA receptor to be activated by high frequency stimulation.

## Discussion

Zinc is a physiologically-relevant ion for activity-dependent long-term regulation of synaptic plasticity [35]. In the present study, SCH-CA1 LTP evoked by TBS was significantly enhanced by 1μM ZnCl_2,_ in agreement with prior findings, though the mechanism by which zinc enhances LTP remains largely unclear [36].

In addition to exogenously applying Zn^2+^, we tested the requirement for endogenous Zn^2+^ on LTP, using the high affinity chelator for extracellular Zn^2+^, CaEDTA [37–40]. In our experiments, chelation of endogenous Zn^2+^ from the synapse resulted in a reduction in the magnitude of LTP, indicating that extracellular ambient concentrations of Zn^2+^ are necessary for full expression of LTP [8,10, 41]. TPEN, another potent Zn^2+^ chelator capable of chelating both extracellular and intracellular Zn^2+^, also blocked LTP to a greater extent than CaEDTA. These data strongly suggest that intracellular and extracellular Zn^2+^ are both involved in mechanisms of LTP.

Exogenously applied zinc did not alter baseline SCH-evoked fEPSPs, which are mediated predominantly by AMPA and GABA receptor conductances, with little contribution from NMDARs subject due to voltage-dependent Mg^2+^ block [42]. We saw no significant changes in fEPSPS or paired-pulse response profiles after prolonged exposure to Zn^2+^, suggesting that micromolar concentrations of Zn^2+^ do not significantly affect AMPA receptor conductance, alter presynaptic release properties at glutamatergic SCH terminals, or have other divalent cation effects that alter excitability. The effects of Zn^2+^ on CA1 synapses appear to be specific, probably localized to postsynaptic CA1 pyramidal neuron conductances (such as NMDA receptor-gated responses) necessary for inducing LTP. In further support of this hypothesis, the NMDAR antagonist D-AP5 blocked both induction of control LTP and its enhancement by 1μM ZnCl_2_, in field CA1. Taken together, our data suggest that ZnCl_2_ is likely to be influencing processes involved in stimulus-evoked synaptic potentiation via postsynaptic NMDARs.

ZnCl_2_ (1μM) significantly increased isolated NMDAR fEPSPs and NMDA evoked whole-cell currents. There are a number of possible causes of this, including but not limited to, an increase in NMDAR channel open time, the frequency of opening and/or closing, and an increase in the number of NMDA receptors in the synapse. Interestingly, the increase in current area may indicate an increase in the number of NR2B containing NMDARs, which have slower activation kinetics [29], or an increased activation of NMDARs containing NR2B subunits (a subunit known to interact with Zn2+), possibly via phosphorylation/dephosphorylation mechanisms [19, 43]. Our data, though not definitive proof of NR2B subunit selectivity are highly suggestive of NR2B involvement and prompted further investigation.

Ro25-6981, an NR2B-selective NMDAR inhibitor, blocked the enhancement of NMDA fEPSPs by 1μM ZnCl_2._ Of note, Ro25-6981 binding is to the N-terminal domain on the extracellular surface, where it competes with Zn^2+^ for this binding domain, suggesting a possible mechanism interfering with enhancement of NMDA current by 1μM ZnCl_2_ [25]. Alternatively, Zn^2+^ may act via an intracellular route that is still susceptible to blockade by Ro25-6981. The importance of intracellular Zn^2+^ in promoting LTP is suggested by the effects of the cell-permeant Zn^2+^ chelator, TPEN, in blocking effects of Zn^2+^. Our data show that inhibition of NR2B-containing NMDAR abrogates the Zn^2+^ mediated increase in NMDA fEPSPs, indicating that Zn^2+^ requires NR2B containing NMDA receptors to mediate an increase in NMDA receptor current.

The next step in our experiments was to see if NR2B-containing NMDARs subunits were required to enhance TBS induced LTP at CA1 synapses. Ro25-6981 did not significantly alter the magnitude of LTP when bath applied alone, which suggests that NR2B-containing NMDAR do not contribute substantially to control LTP elicited by TBS stimulation. This is consistent with literature suggesting that hippocampal NR2B subunits play a more predominate role in the induction of LTP in younger mice, particularly during development [44]. However, low micromolar [Zn^2+^] was unable to enhance LTP in the presence of Ro25-6981, confirming a requirement of NR2B containing NMDA receptor activation for the enhancement of SCH-CA1 LTP by Zn^2+^. Thus, Zn^2+^ enhancement of LTP may be mediated by the up-regulation of NR2B-containing NMDARs to contribute to induction of LTP in adult rat hippocampus.

Zinc modulation of NMDA receptors could be the result of Zn^2+^ crossing the membrane through other channels and activating intracellular signaling molecules [32, 45]. A likely zinc target is the Src family of tyrosine kinases (SFK), which has been shown to modulate NMDA receptor activity via phosphorylation of specific tyrosine residues in the presence of zinc [12,13]. In the present study, PP2, a general SFK inhibitor, inhibited Zn^2+^ mediated NMDA fEPSP. It appears that the timing of PP2 pretreatment was important; if ZnCl_2_ and PP2 were co-applied with no initial pre-treatment of PP2, NMDA fEPSPs were still enhanced, while pre-treatment with PP2 blocked the actions of Zn^2+^. This could be explained by more rapid transmembrane penetration of the charged Zn^2+^ compared to the larger PP2. Nevertheless, our data do suggest that Zn^2+^ acts via NR2B SFK phosphorylation to enhance NMDA fEPSP activity.

During induction of stimulus-evoked LTP, there are crucial phosphorylation cascades that are activated, resulting in phosphorylation at multiple sites (serine, threonine, tyrosine) on many proteins, and multiple levels of direct and indirect modulation of AMPAR and NMDAR transmission. In our experiments, PP2 did not alter TBS-induced LTP at SCH-CA1 synapses, suggesting that SFK activity is not necessary for the induction and maintenance of this form of LTP. However, PP2 did specifically block the increase in LTP elicited by 1μM ZnCl_2,_ supporting a requirement for SFK activation in modulation of LTP by Zn^2+^.

To further elucidate mechanisms by which PP2 inhibited Zn^2+^ enhancement of LTP, we examined the role of phosphorylation at the Y1472. Y1472 has been implicated as a key phosphorylation site of recombinant NR2B-containing NMDAR [13]. Phosphorylation of this site demonstrates increased NMDA receptor current, increased NR2B-containing NMDA receptors at the cell surface, and recruitment of NR2B NMDA receptors to the synapse from extrasynaptic sites following induction of LTP [15, 46,47]. We examined whether Zn^2+^ could alter the phosphorylation of the NR2B receptor subunit at baseline, without any stimulation to the tissue. Thus allowing us to examine whether or not the NR2B subunit was “primed” by phosphorylation at Y1472 before application of high frequency stimulus. Western blot analysis revealed that 1μM ZnCl_2_ did not increase tyrosine phosphorylation of the NR2B subunit at Y1472 prior to TBS. The addition of the inhibitor PP2 had no influence on the basal state phosphorylation at the Y1472 site. Our findings suggest that exposure to Zn^2+^ without stimulation does not result in an increase in phosphorylation at the site Y1472. However, this does not rule out a role for Y1472 phosphorylation in the enhancement of LTP, which could occur with the combination of Zn^2+^ and high-frequency synaptic activation. There could be other sites of phosphorylation that do enhance at baseline or after stimulation which should be explored in this context.

The question remains as to whether Zn^2+^ enhancement of LTP via activation of the SFK pathway is Ca^2+^-dependent, or enhanced due to a direct activation of the signaling pathway that bypasses Ca^2+^. Although NR2A containing NMDARs have been shown to predominantly contribute to LTP in adult mice, NR2B-containing NMDARs in Field CA1 do contribute to calcium transients, suggesting a potential regulatory role in LTP formation that could be up-regulated by Zn^2+^ [48]. Although NR2A and NR2B receptors interact with many of the same signaling molecules, tyrosine phosphorylation of NR2B could uniquely be modulated by the inhibitory scaffolding protein RACK1. RACK1 has been shown to prevent tyrosine phosphorylation of NR2B, resulting in reduced NMDA receptor activity [49]. A possible scenario is that Zn^2+^ may act to release RACK1 from NR2B thus increasing NMDAR activation.

Another possible contributor to Zn^2+^ enhancement of LTP are triheteromeric NMDA receptors. Trihetermoric NMDARs contain two NR1 subunits and both NR2A and NR2B subunits in combination, which, as some studies suggest, may comprise more than 50% of the NMDARs in the synapse in the hippocampus [50]. Their function in the hippocampus has yet to be fully understood, but their contribution to the responsiveness to Zn^2+^ binding as well as to possible differences in pharmacological interaction could provide an alternative site of interaction and modulation. Further examination of the kinetics and functions of triheteromeric NMDA receptors at CA1 synapses in the presence of Zn^2+^ is warranted [51]. As a conjecture, Zn^2+^ may cause a change in the interaction between NR2A and NR2B that favors one subunit over the other in the NMDA receptor complex, changing the mix of NMDARs in the synapse.

## Conclusion

The data presented here support the conclusion that low micromolar zinc enhances TBS-induced LTP of SCH-CA1 synapses through phosphorylation of NR2B subunits of NMDA receptors. Our findings suggests a specific endogenous role for Zn^2+^ as a modulator of LTP, not related to a generalized properties of heavy metals in the brain [47]. Given the differences between physiological synaptic release of Zn^2+^ and exogenously applied Zn^2+^, bath application of exogenous ZnCl_2_ has the potential to interact with an extrasynaptic receptor population with a higher percentage of NR2B-containing NMDAR that is activated by glutamate spillover during high-frequency stimulation. In conclusion, the data presented here suggests that zinc influences activity-dependent synaptic plasticity within a relatively narrow range, the balance of which may be critical for regulation of LTP of synaptic strength underlying learning and memory formation. Dysregulation of zinc homeostasis that results in altered concentrations of Zn^2+^ release may be a key target for therapeutic intervention in a multitude of brain pathologies.

## Supporting information

S1 Fig 0.01μM and 10μM ZnCl_2_ do not significantly alter CA1-LTP. The time course of LTP in untreated control slices (circles) compared to slices treated with 10μM (squares, n = 11) or 0.01μM ZnCl_2_ (triangles, n = 13). Neither 0.01μM nor 10μM ZnCl_2_ significantly altered LTP magnitude compared to controls (P>0.05; 1-way ANOVA).
(TIFF)

S2 Fig 0.01μM and 10μM ZnCl_2_ did not enhance NMDAR fEPSPs. (A)Time course of NMDAR fEPSPs (amplitude) enhanced and isolated by application of Mg^2+^-free ACSF plus 25μM DNQX. ZnCl_2_ was bath applied after 15 minutes of stable baseline. 100nM ZnCl_2_ (squares, n = 4) and 10μM ZnCl_2_ (triangles, n = 6) transiently enhanced NMDAR fEPSPs compared to untreated control slices (circles, n = 7), an effect that had reversed by one hour of application. (Each point mean ± SEM of n recordings). (B) Waveform of NMDA fEPSPs in presence of magnesium at 0’ without zinc and after 60’ zinc exposure demonstrating no effect in the presence of magnesium.
(TIFF)

S3 Fig Zn^2+^ increases charge transfer of Schaffer collateral-evoked NMDAR conductances. This is the time course of NMDA evoked currents for 1μM ZnCl_2_ (circles, n = 5) and NMDA evoked currents for control (circles, n = 6). Baseline was recorded for 10 minutes before application of ZnCl_2._ Bath application of ZnCl_2_ increased the area compared to baseline. (P<0.05; Student’s t-test).
(TIFF)

S4 Fig Ifenprodil and NVP AAM077 blockade of NMDA differentially affects Zn^2+^-mediated enhancement of NMDA receptors. (A) Time course of NMDA fEPSP amplitudes in control slices (dark circles, n = 6) in the presence of 10μM ifenprodil alone, versus slices treated with ifenprodil + 1μM ZnCl_2_ (light circles, n = 2). (B) Histogram demonstrating how ifenprodil inhibited NMDA fEPSPS in the presence of 1μM ZnCl_2_. (C) Time course of NMDA fEPSP amplitudes in control slices (dark trace, n = 6) treated with NVP AAM077 alone, versus slices treated with NVP AAM077 + 1μM ZnCl_2_ (light circles, n = 5).
(TIFF)

S5 Fig PP3 does not inhibit zinc enhancement of CA1 LTP. (A) The time course of LTP in PP2 treated slices (circles) compared to slices treated with PP3 (squares). PP3 did not inhibit LTP magnitude compared to PP2 (*P*<0.05; Student’s t-test). (B) Summary of the normalized slope at 50 minutes after TBS stimulation. Mean ± SEM of fEPSP slopes 50 minutes post-TBS in slices treated with 1μM ZnCl_2_ + PP2 (n = 4) versus slices treated with 1μM ZnCl_2_ + PP3 (n = 3). The two groups were significantly different, with PP2, but not PP3, completely blocking the Zn^2+^ enhancement of LTP (*, *P*<0.05; Student’s t-test).
(TIFF)

S6 Fig Zn^2+^ does not alter basal phosphorylation of NR2B NMDAR subunits at Y1472. A representative western blot. All samples in this figure are from the same animal. The lanes above each other are duplicate aliquots of the same slice. Upper panels are developed with anti NMDAR2B antibody and the lower panels developed with anti PY NMDAR2B antibody. Quantitation of the ECL developed image was performed using Image J software. Quantitative comparisons were performed by normalizing each blot to a control lane and the relative value of the PY signal was divided by the total NMDAR2B signal. Thus a ratio of one is the value of control standard- the PY/NMDA for control is set to 1. The quantization from this study is reported in Fig 6E.
(TIFF)
