# Supplementary figures and images for: Zinc enhances hippocampal long-term potentiation at CA1 synapses through NR2B containing NMDA receptors
Source: PLoS One. 2018 Nov 28;13(11):e0205907. doi: 10.1371/journal.pone.0205907 (PMC6261414; doi:10.1371/journal.pone.0205907)

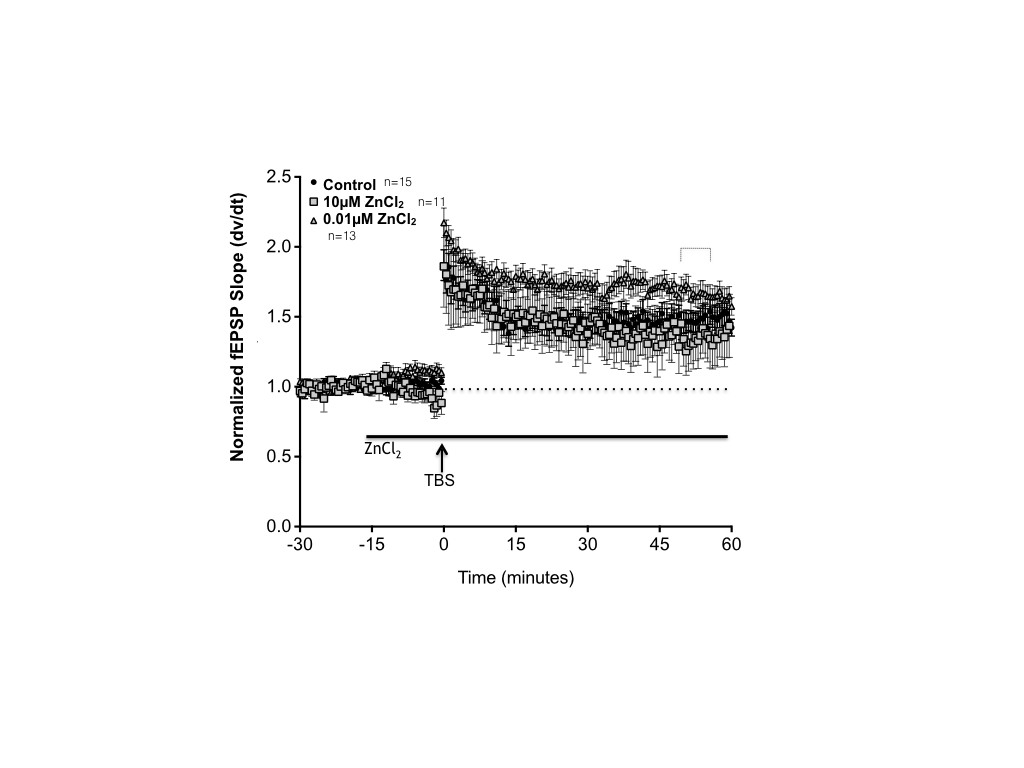

Supplement: S1 Fig — The time course of LTP in untreated control slices (circles) compared to slices treated with 10μM (squares, n = 11) or 0.01μM ZnCl2 (triangles, n = 13). Neither 0.01μM nor 10μM ZnCl2 significantly altered LTP magnitude compared to controls (P>0.05; 1-way ANOVA). (TIFF) [file pone.0205907.s001.tiff]

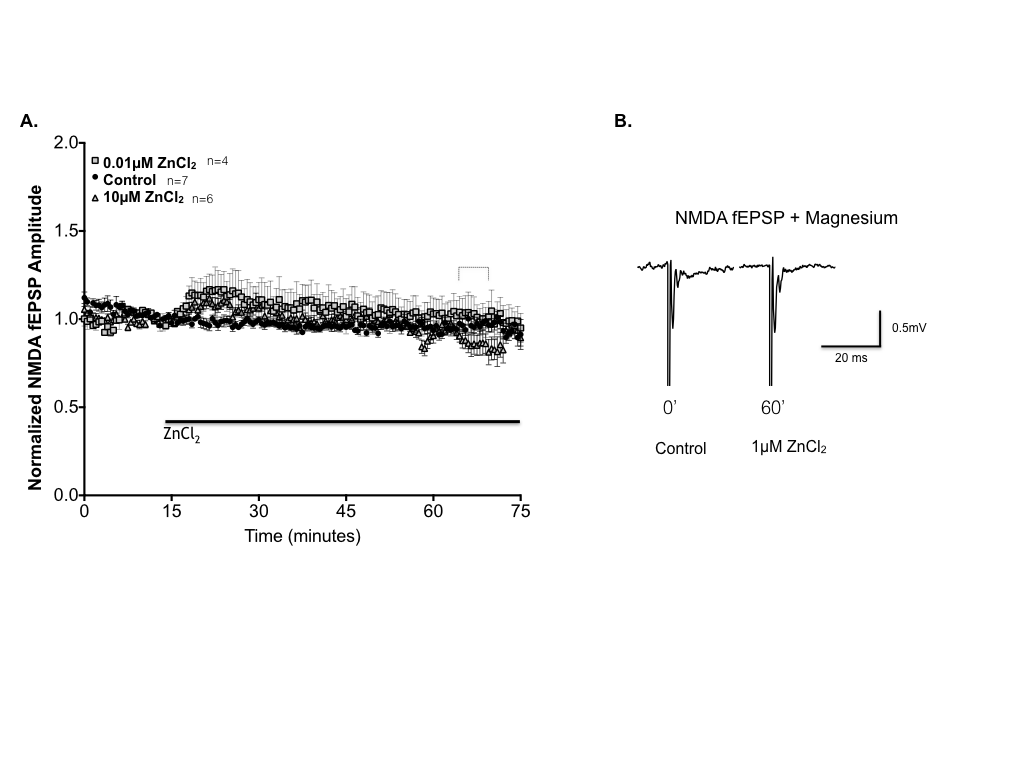

Supplement: S2 Fig — (A)Time course of NMDAR fEPSPs (amplitude) enhanced and isolated by application of Mg2+-free ACSF plus 25μM DNQX. ZnCl2 was bath applied after 15 minutes of stable baseline. 100nM ZnCl2 (squares, n = 4) and 10μM ZnCl2 (triangles, n = 6) transiently enhanced NMDAR fEPSPs compared to untreated control slices (circles, n = 7), an effect that had reversed by one hour of application. (Each point mean ± SEM of n recordings). (B) Waveform of NMDA fEPSPs in presence of magnesium at 0’ without zinc and after 60’ zinc exposure demonstrating no effect in the presence of magnesium. (TIFF) [file pone.0205907.s002.tiff]

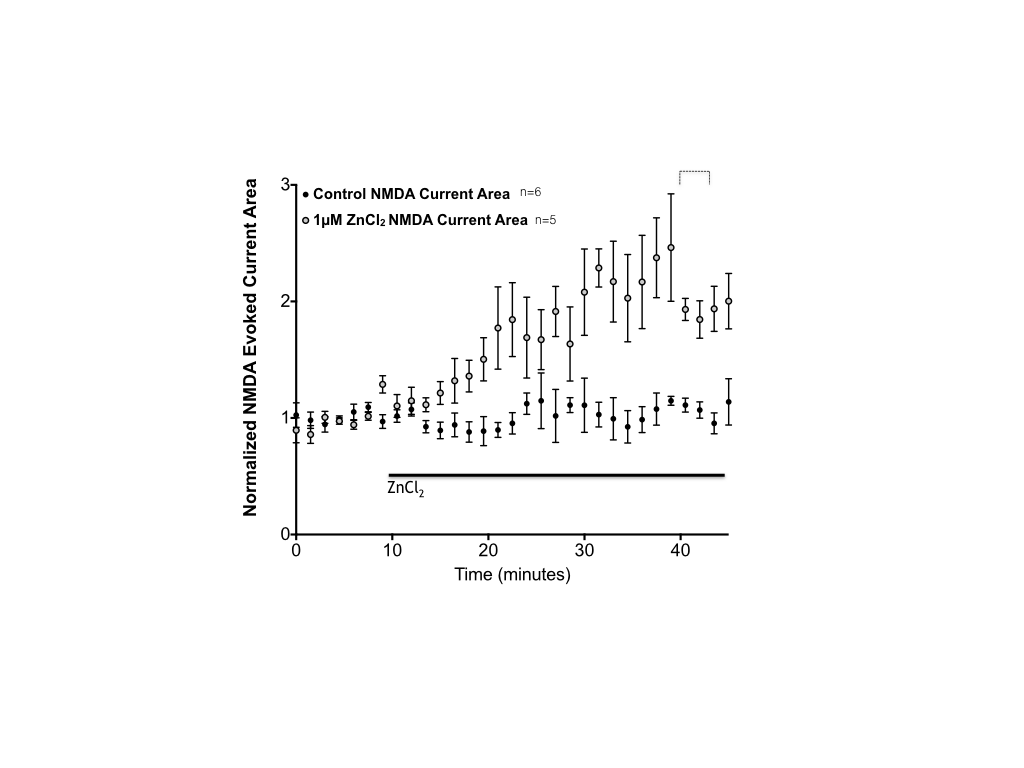

Supplement: S3 Fig — This is the time course of NMDA evoked currents for 1μM ZnCl2 (circles, n = 5) and NMDA evoked currents for control (circles, n = 6). Baseline was recorded for 10 minutes before application of ZnCl2. Bath application of ZnCl2 increased the area compared to baseline. (P<0.05; Student’s t-test). (TIFF) [file pone.0205907.s003.tiff]

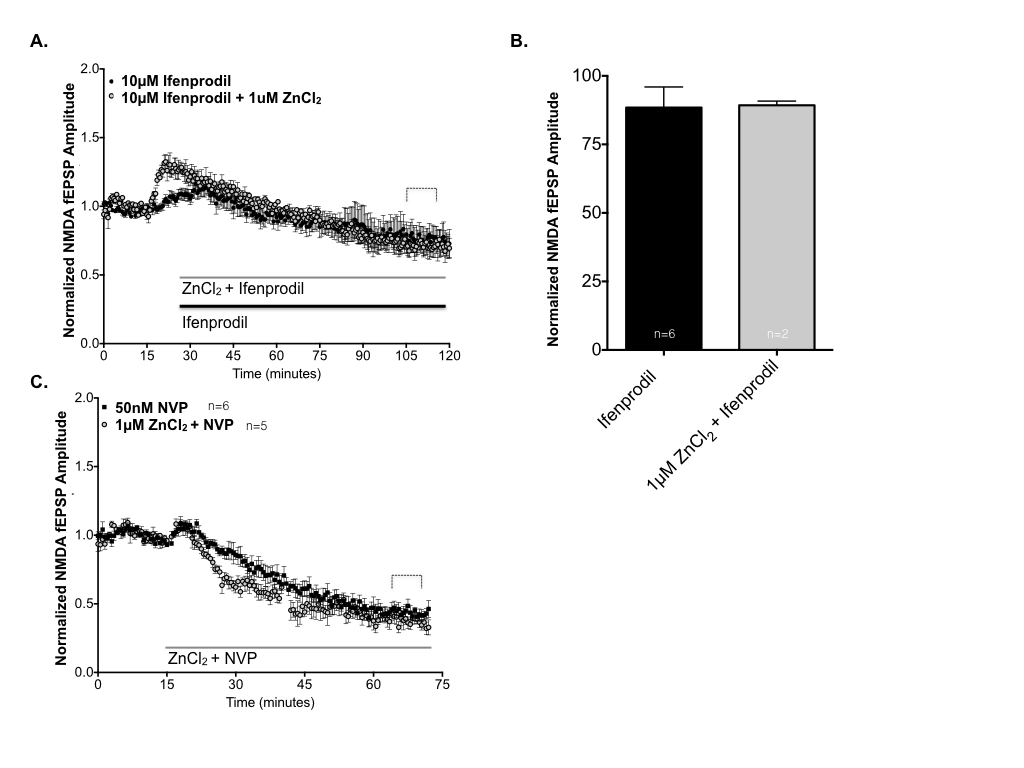

Supplement: S4 Fig — (A) Time course of NMDA fEPSP amplitudes in control slices (dark circles, n = 6) in the presence of 10μM ifenprodil alone, versus slices treated with ifenprodil + 1μM ZnCl2 (light circles, n = 2). (B) Histogram demonstrating how ifenprodil inhibited NMDA fEPSPS in the presence of 1μM ZnCl2. (C) Time course of NMDA fEPSP amplitudes in control slices (dark trace, n = 6) treated with NVP AAM077 alone, versus slices treated with NVP AAM077 + 1μM ZnCl2 (light circles, n = 5). (TIFF) [file pone.0205907.s004.tiff]

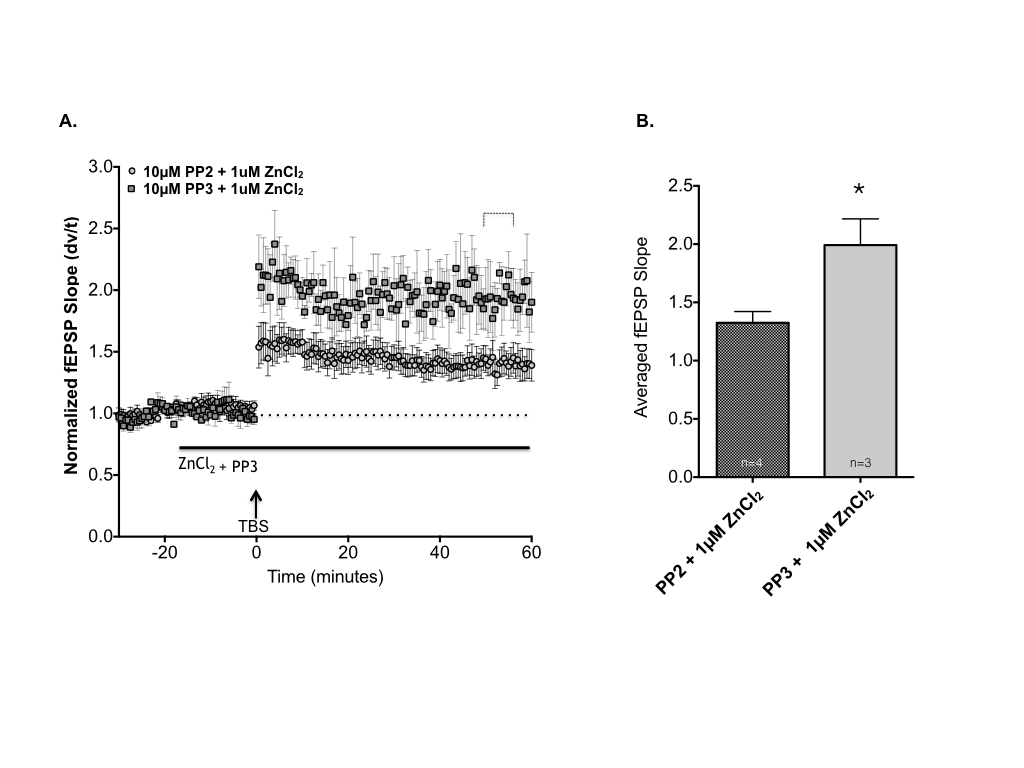

Supplement: S5 Fig — (A) The time course of LTP in PP2 treated slices (circles) compared to slices treated with PP3 (squares). PP3 did not inhibit LTP magnitude compared to PP2 (P<0.05; Student’s t-test). (B) Summary of the normalized slope at 50 minutes after TBS stimulation. Mean ± SEM of fEPSP slopes 50 minutes post-TBS in slices treated with 1μM ZnCl2 + PP2 (n = 4) versus slices treated with 1μM ZnCl2 + PP3 (n = 3). The two groups were significantly different, with PP2, but not PP3, completely blocking the Zn2+ enhancement of LTP (*, P<0.05; Student’s t-test). (TIFF) [file pone.0205907.s005.tiff]

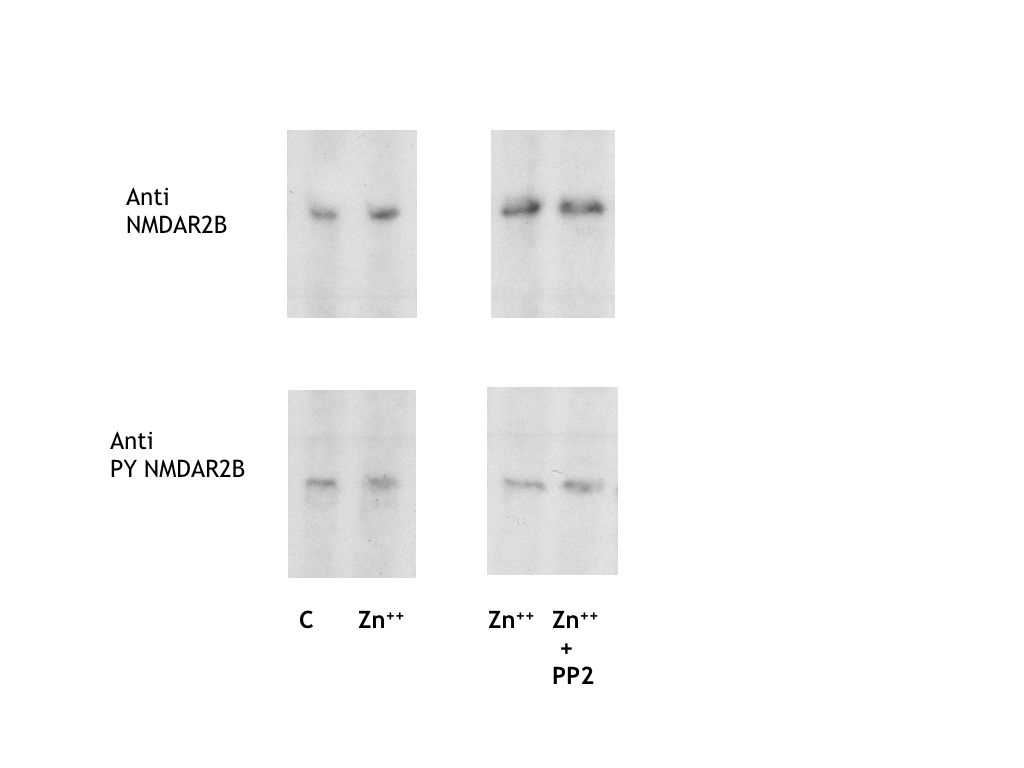

Supplement: S6 Fig — A representative western blot. All samples in this figure are from the same animal. The lanes above each other are duplicate aliquots of the same slice. Upper panels are developed with anti NMDAR2B antibody and the lower panels developed with anti PY NMDAR2B antibody. Quantitation of the ECL developed image was performed using Image J software. Quantitative comparisons were performed by normalizing each blot to a control lane and the relative value of the PY signal was divided by the total NMDAR2B signal. Thus a ratio of one is the value of control standard- the PY/NMDA for control is set to 1. The quantization from this study is reported in Fig 6E. (TIFF) [file pone.0205907.s006.tiff]
